# Peri-operative Outcomes and Survival Following Palliative Gastrectomy for Gastric Cancer: a Systematic Review and Meta-analysis

**DOI:** 10.1007/s12029-020-00519-4

**Published:** 2020-09-22

**Authors:** Joseph Cowling, Bethany Gorman, Afrah Riaz, James R. Bundred, Sivesh K. Kamarajah, Richard P. T. Evans, Pritam Singh, Ewen A. Griffiths

**Affiliations:** 1 College of Medical and Dental Sciences, University of Birmingham, Birmingham, UK; 2 College of Medical and Dental Sciences, University of Leeds, Leeds, UK; 3 Department of Upper GI surgery, Queen Elizabeth Hospital Birmingham, University Hospitals Birmingham NHS FT, Mindelsohn Way, Birmingham, B15 2TH UK; 4 Nottingham Oesophago-Gastric Unit, City Hospital, Hucknall Rd, Nottingham, NG5 1PB UK

**Keywords:** Stomach neoplasms, Gastrectomy, Survival

## Abstract

**Background:**

Many patients with gastric cancer present with late stage disease. Palliative gastrectomy remains a contentious intervention aiming to debulk tumour and prevent or treat complications such as gastric outlet obstruction, perforation and bleeding.

**Methods:**

We conducted a systematic review of the literature for all papers describing palliative resections for gastric cancer and reporting peri-operative or survival outcomes. Data from peri-operative and survival outcomes were meta-analysed using random effects modelling. Survival data from patients undergoing palliative resections, non-resective surgery and palliative chemotherapy were also combined. This study was registered with the PROSPERO database (CRD42019159136).

**Results:**

One hundred and twenty-eight papers which included 58,675 patients contributed data. At 1 year, there was a significantly improved survival in patients who underwent palliative gastrectomy when compared to non-resectional surgery and no treatment. At 2 years following treatment, palliative gastrectomy was associated with significantly improved survival compared to chemotherapy only; however, there was no significant improvement in survival compared to patients who underwent non-resectional surgery after 1 year. Palliative resections were associated with higher rates of overall complications versus non-resectional surgery (OR 2.14; 95% CI, 1.34, 3.46; *p* < 0.001). However, palliative resections were associated with similar peri-operative mortality rates to non-resectional surgery.

**Conclusion:**

Palliative gastrectomy is associated with a small improvement in survival at 1 year when compared to non-resectional surgery and chemotherapy. However, at 2 and 3 years following treatment, survival benefits are less clear. Any survival benefits come at the expense of increased major and overall complications.

**Electronic supplementary material:**

The online version of this article (10.1007/s12029-020-00519-4) contains supplementary material, which is available to authorized users.

## Introduction

Primary gastric cancer (GC) is the fifth most common malignancy worldwide and frequently presents at a late and incurable stage [1]. The majority of patients present with either stage 3 or 4 disease and many will have already developed metastasis [2, 3] with many patients surviving less than a year after initial diagnosis [4, 5]. Although the incidence of GC is declining, there are still over 5000 new diagnoses every year in the UK alone and it continues to be the 3^rd^ biggest cause of cancer-related deaths globally [6–8].

Localised GC is often managed with combined resection and chemotherapy owing to a significant body of evidence which demonstrates its survival benefit compared to surgery alone [9–11]. However, advanced GC is generally regarded as incurable and resection is often not considered owing to the extent of local tumour invasion and/or the presence of distant metastases [12]. Progressive tumour growth means patients are at risk of tumour-related complications such as gastric outlet obstruction, perforation and bleeding, all of which can lead to reduced quality of life, emergency surgery and ultimately a reduction in life span.

Palliative gastrectomy (PG), comprising of either total, subtotal or distal gastrectomy, is recognised as a treatment for alleviating or preventing these complications, yet its use remains a contentious topic owing to the high-risk nature of the procedure and mixed evidence for its survival benefit in advanced GC [13–15].

Previous evidence has not only demonstrated the absence of any survival benefit from PG but has also shown no improvement in quality of life and an increased number of chemotherapy-associated adverse events [14, 15]. The REGATTA trial, the only phase III randomised control trial comparing chemotherapy alone and gastrectomy followed by chemotherapy showed no survival benefit and concluded that palliative gastrectomy in patients with metastatic gastric cancer cannot be justified [14]. Some authors have criticised the REGATTA trial for including large numbers of patients requiring total gastrectomy, using oral rather than intra-venous chemotherapy treatment regimens and grouping patients with different sites of metastatic disease together as these factors could affect the interpretation of the results [16].

There is a growing body of non-randomised evidence suggesting that PG not only provides symptomatic relief but can also extend survival [17–20]. With continued uncertainty surrounding the efficacy of PG in advanced GC, the aim of this systematic review and meta-analysis was to analyse both operative and survival outcomes following palliative gastrectomy for advanced primary gastric cancer.

## Methods

### Search Strategy

**Fig. 1:**
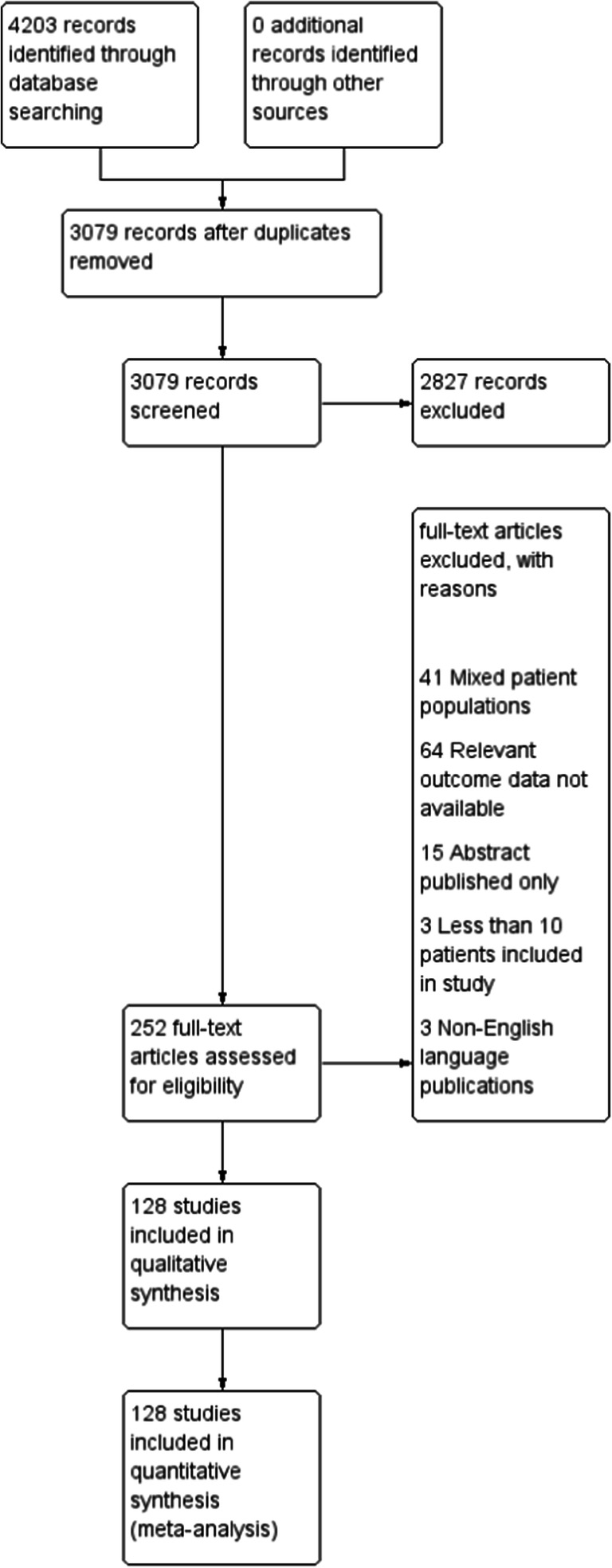
PRISMA diagram of study inclusion

This study was prospectively registered with the PROSPERO database of systematic reviews (CRD42019159136). A systematic literature search was undertaken by one researcher (SK) using the PubMed, EMBASE and Cochrane Library databases on 25^th^ January 2020. Search terms included ‘palliative gastrectomy’ or ‘palliative total gastrectomy’ or ‘palliative subtotal gastrectomy’ or ‘palliative resection’ and ‘stomach neoplasms’ or ‘gastric cancer’ or ‘gastric adenocarcinoma’ or ‘stomach cancer’. Outcomes including ‘post-operative complications’, ‘mortality’, ‘disease free survival’, ‘overall survival’ and ‘quality of life’ were included in the search. Full details of the literature search terms used can be found in Supplementary table 1. The results of the literature search were reported in accordance with the PRISMA guidelines (Fig. 1).

### Inclusion and Exclusion Criteria

Inclusion criteria were (1) studies reporting outcomes following palliative gastrectomy for primary gastric adenocarcinoma and (2) human studies published in the English language. Exclusion criteria were (1) review articles, case reports, letters, editorials and conference abstracts; (2) studies which exclusively report outcomes for oesophagectomy, oesophagogastrectomy, surgical bypass procedures or curative gastrectomy; (3) studies in which outcomes for palliative gastrectomy were combined with the outcomes of other surgical procedures; (4) gastric cancers other than primary adenocarcinoma.

All studies generated by the literature search were screened by three independent reviewers for their relevance based on the title, abstract and study type using the above inclusion/exclusion criteria. All duplications were excluded. In the instance, there was uncertainty about the relevance of a study, the advice was sought of all authors and a final decision was made. Where studies were excluded, the reason for exclusion was verified by a fourth reviewer. For those studies which remained following this initial screening process, full texts were obtained and reviewed in detail by the same three to produce a final list of all included studies.

### Study Outcomes

The primary outcome was overall survival following palliative gastrectomy for primary gastric cancer. Secondary outcomes included overall post-operative complications, major complications, anastomotic leak, pulmonary complications, mortality, overall survival rates (1-, 2-, 3- and 5-year), recurrence-free survival and self-reported quality of life measures.

### Data Extraction

Data was extracted for all included studies by three independent reviewers and any queries were resolved by consensus with all authors. Data was extracted under the following headings: year of publication, study duration, study country, study design, number of study centres, use of comparison groups, overall study sample size, treatment group sample size, stage of gastric cancer, definition of palliative gastrectomy, tumour location, metastasis location, tumour histology, risk factors and chemotherapy use. In addition to extracting data for patients undergoing palliative gastrectomy, where available, data was extracted for other treatment groups under the broad headings of ‘curative gastrectomy’, ‘chemotherapy only’, ‘non-resectional surgery’ and ‘no surgery’. This data was collected to enable a comparison to the main intervention of interest, palliative gastrectomy.

### Assessment of Methodological Quality

Three researchers assessed the methodological quality of all included studies using the Newcastle-Ottawa Quality Assessment Scale (NOS) for all comparative cohort studies. This score was omitted in the instance that a study was a non-comparative cohort study, for which the NOS is not valid. The overall grading of each study is given in results supplementary table 1.

### Statistical Analysis

This systematic review and meta-analysis was conducted in accordance with the recommendations of the Cochrane Library and MOOSE guidelines [15]. For categorical variables, analysis was performed by calculating the odds ratio (OR). For survival analysis, relative risk (RR) statistics were calculated. Random effects modelling, using the DerSimonian-Laird method was used for the meta-analysis of outcomes. Heterogeneity between studies was assessed using the *I*_2_ value in order to determine the degree of variation not attributable to chance alone. *I*_2_ values were considered to represent low, moderate and high degrees of heterogeneity where values were < 25%, 25–75%, and > 75%, respectively. Assessment of small study bias was carried out by visual assessment of funnel plots and egger regressions. Statistical significance was considered when *p* < 0.05. Statistical analyses were performed using R statistical software (R version 3.5.2, R Foundation for Statistical Computing, Vienna, Austria).

## Results

### Study Characteristics

The literature search identified 128 studies reported according to the PRISMA guidelines as shown in Fig. 1. Studies identified were from North America (*n* = 14), South America (*n* = 7), Australasia (*n* = 72) and Europe (*n* = 35). The majority of studies were retrospective cohort studies (*n* = 123), with the remainder prospective cohort studies (*n* = 3) and RCTs (*n* = 2). Sixty-one studies identified were reported after 2010, the remaining 67 studies before 2010. Of the studies that reported on either clinical or pathological tumour stage, 41 of 91 studies consisted entirely of patients with T stage 4 disease. On average across 91 studies reporting the percentage of patients with T stage 4 disease, 68.6% of patients had T stage 4 disease. There was considerable variation in whether resections were defined as palliative due to the advanced T stage of the primary tumour or due to distant metastasis (Table 1). Across studies containing a proportion of patients with metastatic disease, 9 studies of 93 included only patients with lymph node metastases, whilst 84 included patients with a mixture of metastases sites. Of these, 41 of 84 studies included patients with liver metastases, 14 studies included patients with lung metastases and 40 included patients with peritoneal metastases.

**Table 1:** Demographics of the included studies

Study (Ref.)	Study year	Study country	Centre number	Study type	Total patients	Number TIV	Location	Mets	Histology
Lulu 1974 [21]	1954–1970	USA	Single	RCS	100		Mixed	Distant	AC
Zacho 1974 [22]	1949–1969	Denmark	Single	RCS	776		Mixed	Distant	AC
Zwaveling 1976 [23]	1958–1972	Netherlands	Single	RCS	217				AC
Nelson 1982 [24]	1970–1975	Australia	Single	RCS	229		Mixed		AC
Yap 1982 [25]	1950–1974	USA	Single	RCS	465				AC
Choi 1982 [26]	1974–1979	Hong Kong	Single	RCS	119		Mixed		AC
Meijer 1983 [27]	1965–1981	Netherlands	Single	RCS	204	204		Distant	AC
Yan 1985 [28]	1958–1982	USA	Single	RCS	196	66			AC
Cunningham 1987 [29]	1974–1984	UK	Single	RCS	328		Mixed		
Bozzetti 1987 [30]	1965–1980	Italy	Single	RCS	294			Distant	AC
de Calan 1988 [31]	1968–1983	France	Single	RCS	91	8	Proximal third	Distant	AC
Butler 1989 [32]	1979–1988	USA	Single	RCS	27	14		Distant	AC
Haugstvedt 1989 [18]	1982–1984	Norway	Multiple	PCS	1165	460			AC
Carmalt 1990 [33]	1974–1987	Australia	Single	RCS	511		Mixed		AC
Habu 1990 [34]	1972–1986	USA	Single	RCS	196	126		Distant	
Nakajima 1991 [35]	1846–1988	Tokyo	Single	RCS	811	811			
Yonemura 1991 [36]	1978–1988	Japan	Single	RCS	76	76		Lymph node	AC
Monson 1991 [37]	1980–1989	USA	Single	RCS	53	17		Distant	AC
Maehara 1992 [1, 38]	1965–1985	Japan	Single	RCS	194	194	Mixed	Distant	AC, undiff
Maehara 1992 [39]	1965–1985	Japan	Single	RCS	1500	1116	Mixed	Distant	AC
Huguier 1992 [40]	1970–1988	France	Single	RCS	197				AC
Maehara 1992 [41]	1965–1985	Japan	Single	RCS	1352		Mixed	Distant	AC, undiff
Baba 1992 [42]	1975–1980	Japan	Single	RCS	119	105	Mixed	Distant	AC, undiff
Geoghegan 1993 [43]	1982–1986	UK	Single	RCS	114			Distant	AC
Ti 1993 [44]	1979–1992	Singapore	Single	RCS	160	88	Antrum and cardia		AC
Crookes 1995 [45]	1988–1993	USA	Single	RCS	204	120	Mixed	Distant	
Chow 1995 [46]	1985–1990	Hong Kong	Single	RCS	38		Mixed	Distant	AC, undiff
Arak 1996 [47]	1983–1987	Finland	Single	RCS	203		Mixed		AC
Saito 1996 [48]	1964–1987	Japan	Single	RCS	116	116		Distant	
Cenitagoya 1998 [49]	1982–1990	Chile	Single	RCS	134		Mixed		
Kikuchi 1998 [50]	1971–1990	Japan	Multiple	RCS	122		Mixed	Distant	
Sanchez-Bueno 1998 [51]	1979–1994	Spain	Single	RCS	297	51	Mixed		AC
Piso 1998 [52]	1986–1997	Germany	Single	RCS	64	44	Mixed	Distant	AC
Ouchi 1998 [15]	1990–1996	Japan	Single	RCS	95	62		Distant	
Piso 1998 [53]	1986–1997	Germany	Single	RCS	33	16	Mixed	Distant	AC
Lo 1999 [54]	1988–1993	Taiwan	Single	RCS	1642	747			
Doglietto 1999 [55]	1981–1995	Italy	Single	RCS	305	305	Mixed	Distant	AC
Llanos 1999 [56]	1975–1993	Chile	Single	RCS			Mixed		AC
Saidi 1999 [57]	1988–1996	Iran	Single	RCS	70	49	Proximal half		
Doglietto 2000 [58]	1981–1995	Italy		RCS	639	305	Mixed	Distant	AC, undiff
Ikeguchi 2001 [59]	1985–1996	Japan	Single	RCS	324	64		Distant	
Hanazaki 2001 [60]	1988–1996	Japan	Single	RCS	184	145	Mixed	Distant	AC
Dhar 2001 [61]	1980–1998	Japan	Single	RCS	150	150			AC, undiff
Fujisaki 2001 [62]	1984–1998	Japan	Single	RCS	43	43		Distant	
Bonenkamp 2001 [63]	1989–1993	Denmark	Single	RCS	285			Lymph node	AC
Liu 2002 [64]	1995–1998	USA	Single	RCS	57		Mixed	Lymph node	AC
Wang 2002 [65]	1994–2000	Taiwan	Single	RCS	415	415	Mixed	Distant	AC
Collard 2003 [66]	2003–2008	Belgium	Single	RCS	216	12	Mixed	Lymph node	AC
Yoshikawa 2003 [67]	1989–2000	Japan	Single	RCS	100	100		Distant	
Gill 2003 [68]	1978–1997	Canada	Single	RCS	2043		Mixed		AC
Kobayashi 2004 [69]	1193–2000	Japan	Single	RCS	82	40		Distant	
Moriwaki 2004 [70]	1981–2004	Japan	Single	RCS	382	382			
Kahlke 2004 [71]	1992–2001	Germany	Single	RCS	169	169	Mixed	Distant	AC, undiff
Medina-Franco 2004 [17]	1995–2000	Mexico	Single	RCS	76		Mixed	Lymph node	AC
Zhang 2004 [72]	1972–2000	China	Single	RCS	2613	622	Mixed	Distant	AC
Gorbunov 2005 [73]	1990–1997	Czech Republic	Multiple	RCS	283	90	Mixed	Lymph node	AC
Kunisaki 2005 [74]	1980–1999	Japan	Single	RCS	183	112	Mixed	Distant	AC
Saidi 2005 [75]	1990–2000	USA	Multiple	RCS	105	105	Mixed	Distant	AC
Alici 2006 [76]	1999–2002	Turkey	Single	RCS	138	138	Mixed	Distant	AC
Samarasam 2006 [77]	1999–2003	India	Single	PCS	151	117		Distant	AC
Onate-Ocana 2007 [78]	1987–2005	Mexico	Single	RCS	132	113	Mixed		AC
Nazli 2007 [79]	1997–2004	Turkey	Single	RCS	74	74	Mixed		AC
Lim 2007 [80]	1989–2001	USA	Single	RCS	63	63	Mixed	Distant	AC
Lello 2007 [81]	1984–2004	Norway	Single	RCS	356	164	Mixed		AC
Mizutani 2007 [82]	1992–2004	Japan	Single	RCS	26	26		Distant	
Nazli 2007 [83]	1997–2004	Turkey	Single	RCS	121	74	Mixed	Distant	AC
Kim 2007 [84]	1986–2000	South Korea	Single	RCS	630	214	Mixed	Distant	AC
Pacelli 2008 [85]	1981–2005	Italy	Single	RCS	400	88	Mixed		AC
Du 2008 [86]	2005–2007	China	Single	RCS	43	43	Mixed	Distant	AC
Lin 2008 [87]	1994–2001	China	Single	RCS	389	389		Distant	AC, undiff
Park 2009 [88]	1996–2005	Korea	Single	RCS	128	12	Mixed	Distant	AC
Lupascu 2010 [89]	2003–2008	Romania	Single	RCS	140	140	Mixed	Distant	AC
Huang 2010 [90]	1988–2008	Taiwan	Single	RCS	2678	166	Mixed	Distant	AC
Hioki 2010 [91]	1993–2004	Japan	Single	RCS	101	101	Mixed	Distant	AC, undiff
Sah 2010 [92]	NS	China	Single	RCS	1639	398	Mixed		
Ozer 2010 [93]	2002 - 2007	Turkey	Single	RCS	549	218	Mixed	Distant	AC
Li 2010 [94]	1992–2002	China	Single	RCS	253	51		Distant	AC, undiff
Xue 2010 [95]	1993–2004	China	Single	RCS	630	630	Mixed	Lymph node	AC
Turanli 2010 [96]	2005–2008	Turkey	Single	RCS	62	62	Mixed	Distant	AC
Schauer 2011 [97]	2011	Germany	Single	RCS	120	38	Mixed	Distant	AC
Al-Amawi 2011 [98]	1998–2009	Poland	Single	RCS	105	105	Mixed	Distant	AC
Tanizawa 2011 [99]	2002–2009	Japan	Single	RCS	18			Distant	
Zhang 2011 [100]	1991–2005	China	Single	RSC	1171	529	Proximal	Distant	AC, undiff
Izuishi 2011 [101]	1984–2008	Japan	Single	RCS	121	121	Mixed	Distant	
Lai 2011 [102]	1988–2009	Taiwan	Single	RCS	295	195	Mixed	Lymph node	AC
Miki 2012 [103]	2012	Japan	Single	RCS	50	40		Distant	AC
Kokkola 2012 [104]	2000–2009	Finland	Single	RCS	55	55		Distant	AC
Shim 2012 [105]	1989–2005	Korea	Single	RCS	278		Mixed	Distant	AC, undiff
Alonso-Larraga 2012 [106]	2005–2010	Mexico	Single	RCS	113	113	Antrum		AC
Tokunaga 2012 [107]	2002–2008	Japan	Single	RCS	148			Distant	AC
Amaral 2012 [108]	1998–2007	Portugal	Single	RCS	155	59	Mixed		AC
Naka 2012 [109]	1991–2007	Japan	Single	RCS	233			Distant	
Chang 2012 [110]	1999–2004	South Korea	Single	RCS	257		Mixed	Distant	
Kang 2013 [111]	2002–2010	Taiwan	Single	RCS	172	172		Distant	AC
Keranen 2013 [112]	1999–2010	Finland	Single	RCS	97	6		Distant	AC
He 2013 [113]	2008–2012	China	Single	RCS	737	224	Mixed	Distant	AC
Ikeguchi 2013 [114]	2003–2010	Japan	Single	RCS	96	96			
Xia 2014 [115]	2014	China	Single	RCS	119	115	Mixed	Distant	AC
Kwon 2014 [116]	1999–2009	Korea	Single	RCS	769	228	Mixed	Distant	AC
Zeeneldin 2014 [117]	2003–2007	Egypt	Single	RCS	168	58	Mixed	Distant	AC
Zeng 2014 [118]	2004–2010	China	Multiple	RCS	533	41	Mixed		AC
Jeong 2014 [119]	2004–2011	South Korea	Single	RCS	197	142	Mixed	Distant	
Kim 2014 [120]	2003–2012	Korea	Single	RCS	43	8			AC, undiff
Da Costa 2015 [121]	1988–2011	Brazil	Single	RCS	413		Mixed	Lymph node	AC
Matsumoto 2015 [122]	2002–2011	Japan	Single	RCS	45		Mixed	Distant	AC
Yao 2015 [123]	2003–2010	China	Single	RCS	49	49	Mixed	Distant	
Yang 2015 [124]	2006–2013	China	Not specified	RCS	267		Mixed	Distant	AC
Ebinger 2016 [19]	1998–2009	USA	Multiple	RCS	8249	8249	Mixed		
Dong 2016 [125]	2002–2012	China	Single	RCS	47	47	Mixed		
Coimbra 2016 [126]	1988–2012	Brazil	Single	RCS	179	179	Mixed		AC
Chiu 2016 [127]	2008–2012	China	Single	RCS	173	173			AC
Fujitani 2016 [14]	2016	Multiple	Multiple	RCT	89		Mixed	Distant	AC
Musri 2016 [128]	2008–2015	Turkey	Single	RCS	288	288		Distant	AC
Ikeguchi 2016 [129]	2003–2012	Japan	Single	RCS	78	78		Distant	AC
Nie 2016 [130]	2000–2014	China	Multiple	RCS	371	371	Mixed	Distant	AC
Al-Batran 2017 [12]	2018	Germany	Multiple	RCT	238	238	Mixed	Distant	AC
Tokunaga 2016 [131]	2002–2011	Japan	Single	RCS	137		Mixed		AC, undiff
Fujitani 2017 [132]	NS	Japan	Multiple	PCS	104	104		Distant	AC
Hsu 2017 [133]	2000–2010	Taiwan	Single	RCS	333	333	Mixed	Distant	AC
Fornaro 2017 [134]	2002–2015	Italy	Multiple	RCS	513		Mixed	Distant	AC
Yuan 2017 [135]	2000–2014	China	Multiple	RCS	201		Mixed	Distant	AC
Fukuchi 2018 [136]	2005–2017	Japan	Single	RCS	94	15	Mixed	Distant	
Warschkow 2018 [20]	2017	USA	Multiple	RCS	7026	7026	Mixed	Distant	AC
Picado 2018 [137]	2004–2014	USA	Multiple	RCS	3175	260	Mixed	Distant	AC
Yuan 2018 [138]	2006–2014	China	Single	RCS	384		Mixed	Distant	AC
Yang 2019 [139]	2004–2013	China	Single	RCS	80		Mixed	Distant	AC
Omori 2019 [140]	2002–2014	Japan	Single	RCS	40		Mixed	Distant	AC
Matsubara 2019 [141]	2004–2015	Japan	Single	RCS	81		Mixed	Distant	AC

*Ref*, reference; *Number TIV*, number of patients with T stage 4 tumours; *AC*, adenocarinoma; *Locatio*n, primary tumour localisation within the stomach; *RCS*, retrospective cohort study; *RCT*, randomised controlled trial

### Reporting Standards and Methodological Quality

Study quality was assessed using NOS, median 8, ranging between 5 and 9, indicating generally high quality cohort studies (Supplementary Table 1). A summary of studies reporting the impact of intervention type on morbidity and mortality is provided in Table 2.

**Table 2:** Differences in short post-operative outcomes comparing non-resectional procedures and curative intent resections to palliative surgery

	*N*	Odds ratio	Confidence intervals	*p* value
Palliative gastrectomy versus non-resectional procedures				
Overall complications	*15*	2.15	1.34–3.46	< 0.001
Major complications	*2*	3.41	1.42–8.20	0.01
Anastomotic leak	*11*	2.35	1.14–4.84	0.02
Peri-operative mortality	*19*	1.10	0.73–1.66	0.66
Palliative gastrectomy versus curative intent resection				
Overall complications	*17*	1.46	1.18–1.79	< 0.001
Major complications	*9*	1.51	0.87–2.62	0.12
Anastomotic leak	*13*	1.01	0.56–1.85	0.98
Peri-operative mortality	*29*	1.89	1.34–2.65	< 0.001
Palliative gastrectomy versus non-resectional procedures (published post-2010)				
Overall complications	*8*	1.493	1.043–2.138	< 0.001
Major complications	*2*	3.41	1.42–8.20	0.01
Anastomotic leak	*6*	2.311	0.653–8.175	0.194
Peri-operative mortality	*3*	0.361	0.082–1.59	0.178
Palliative gastrectomy versus curative intent resection (published post-2010)				
Overall complications	*6*	1.536	1.013–2.328	0.043
Major complications	*2*	1.294	0.392–4.272	0.672
Anastomotic leak	*5*	0.789	0.212–2.915	0.724
Peri-operative mortality	*11*	1.397	0.696–2.821	0.348

### Peri-operative Outcomes

#### Overall Complications

Fifteen studies reported data on overall complications comparing patients undergoing palliative surgery compared to non-resectional procedures. Palliative gastrectomy was associated with an increase in overall complications compared to non-resectional surgery (OR 2.14; 95% CI, 1.34, 3.46; *p* < 0.001; *I*_2_ = 46%) (Table 3). Egger regression analysis suggested a significant publication bias (*p* = 0.004), with a Duval and tweedie imputed OR and 95% CI, 1.43 (0.81, 2.54). Seventeen studies reported data on overall complications comparing palliative surgery to curative intent surgery. Palliative surgery was associated with an increase in overall complications compared to curative surgery (OR 1.46; 95% CI, 1.18, 1.79; *p* < 0.001; *I*_2_ = 47%). No significant publication bias was identified through egger regression testing (*p* = 0.871).

**Table 3:** Relative risk and 95% confidence intervals of different treatment strategies versus palliative gastrectomy at 1-, 2-, 3- and 5-year survival

	*N*	RR	95% CI	*p*	*I*_2_
1-year survival					
Palliative gastrectomy vs. chemotherapy only	5	0.734	0.559–0.963	0.0256	81%
Palliative gastrectomy vs. non-resectional procedures	7	0.421	0.197–0.909	0.0435	82%
Palliative gastrectomy vs. no intervention	8	0.381	0.176–0.827	0.0147	91%
2-year survival					
Palliative gastrectomy vs. chemotherapy only	6	0.508	0.352–0.744	0.040	81%
Palliative gastrectomy vs. non-resectional procedures	5	0.432	0.150–1.194	0.4434	85%
Palliative gastrectomy vs. no intervention	6	0.277	0.239–0.326	< 0.001	0%
3-year survival					
Palliative gastrectomy vs. chemotherapy only	4	0.578	0.298-1.07	0.2285	70%
Palliative gastrectomy vs. non-resectional procedures	1	-	-	-	-
Palliative gastrectomy vs. no intervention	3	0.225	0.181-0.284	< 0.001	0%
Papers published post-2010 subgroup					
1-year survival					
Palliative gastrectomy vs. chemotherapy only	5	0.734	0.559-0.963	0.0256	81%
Palliative gastrectomy vs. non-resectional procedures	1	-	-	-	-
Palliative gastrectomy vs. no intervention	1	-	-	-	-
2-year survival					
Palliative gastrectomy vs. chemotherapy only	6	0.508	0.347-0.742	< 0.001	81%
Palliative gastrectomy vs. non-resectional procedures	1	-	-	-	-
Palliative gastrectomy vs. no intervention	2	1.101	0.407–2.974	0.562	0%
3-year survival					
Palliative gastrectomy vs. chemotherapy only	5	0.567	0.299–1.074	0.0816	54%
Palliative gastrectomy vs. non-resectional procedures	1	-	-	-	-
Palliative gastrectomy vs. no intervention	1	-	-	-	-

#### Major Complications

Two studies reported data on major complications comparing patients undergoing palliative gastrectomy compared to non-resectional procedures. Palliative surgery was associated with an increase in major complications compared to non-resectional surgery (OR 3.41; 95% CI, 1.42, 8.20; *p* < 0.001; *I*_2_ = 0%) (Table 3). Insufficient data were available for egger regression testing. Nine studies reported data on overall complications comparing palliative surgery to curative intent surgery. Palliative surgery was associated with an increase in major complications compared to curative surgery (OR 1.51; 95% CI, 0.87, 2.52; *p* = 0.12; *I*_2_ = 84%). No significant publication bias was identified through egger regression testing (*p* = 0.702).

#### Anastomotic Leak

Eleven studies reported data on anastomotic leak comparing patients undergoing palliative surgery compared to non-resectional procedures. Palliative Surgery was associated with an increase in anastomotic leak compared to non-resectional surgery (OR 2.35; 95% CI, 1.14, 4.84; *p* = 0.02; *I*_2_ = 0%) (Table 3). Egger regression analysis suggested an insignificant publication bias (*p* = 0.654). Thirteen studies reported data on anastomotic leak comparing palliative surgery to curative intent surgery. Palliative surgery was associated with similar rates of anastomotic leak compared to curative surgery (OR 1.01; 95% CI, 0.56, 1.42; *p* = 0.98; *I*_2_ = 71%). No significant publication bias was identified through egger regression testing (*p* = 0.945).

#### Early Post-operative Mortality

Nineeen studies reported data on early post-operative mortality comparing patients undergoing palliative surgery compared to non-resectional procedures. Palliative surgery was not associated with a significant increase in early post-operative mortality compared to non-resectional surgery (OR 1.10; 95% CI, 0.73, 1.66; *p* = 0.66; *I*_2_ = 21%). Egger regression analysis suggested an insignificant publication bias (*p* = 0.495). Twenty-nine studies reported data on early post-operative mortality comparing palliative surgery to curative intent surgery. Palliative surgery was associated with an increase in early post-operative mortality compared to curative surgery (OR 1.89; 95% CI, 1.34, 2.65; *p* = 0.98; *I*_2_ = 43%). No significant publication bias was identified through egger regression testing (*p* = 0.673).

### Long-term Survival

#### 1-Year Survival

Twenty studies reported numbers surviving at 1 year following palliative surgery, non-resectional surgery, chemotherapy or no treatment. Palliative surgery was associated with an improved 1-year survival compared to non-resectional surgery (RR 0.421, 0.197–0.909; *p* = 0.044), chemotherapy (RR 0.734, 0.575–0.963; *p* = 0.026) and no treatment (OR 0.381, 0.176–0.827; *p* = 0.015) (Table 4).

**Table 4:** Relative risk and 95% confidence intervals of different treatment strategies versus palliative gastrectomy at 1-, 2-, - 3- and 5-year survival

	*N*	RR	95% CI	*p*	*I*_2_
1-year survival					
Palliative gastrectomy vs. chemotherapy only	5	0.734	0.559–0.963	0.0256	81%
Palliative gastrectomy vs. non-resectional procedures	7	0.421	0.197–0.909	0.0435	82%
Palliative gastrectomy vs. no intervention	8	0.381	0.176–0.827	0.0147	91%
2-year survival					
Palliative gastrectomy vs. chemotherapy only	6	0.508	0.252–0.997	0.045	81%
Palliative gastrectomy vs. non-resectional procedures	5	0.442	0.071–2.697	0.4434	85%
Palliative gastrectomy vs. no intervention	6	0.277	0.239–0.326	< 0.001	0%
3-year survival					
Palliative gastrectomy vs. chemotherapy only	4	0.578	0.298–1.12	0.2285	70%
Palliative gastrectomy vs. non-resectional procedures	1	-	-	-	-
Palliative gastrectomy vs. no intervention	3	0.225	0.181-0.284	< 0.001	0%
Papers published post-2010 subgroup					
1-year survival					
Palliative gastrectomy vs. chemotherapy only	5	0.734	0.559–0.963	0.0256	81%
Palliative gastrectomy vs. non-resectional procedures	1	-	-	-	-
Palliative gastrectomy vs. no intervention	1	-	-	-	-
2-year survival					
Palliative gastrectomy vs. chemotherapy only	6	0.508	0.347-0.742	< 0.001	81%
Palliative gastrectomy vs. non-resectional procedures	1	-	-	-	-
Palliative gastrectomy vs. no intervention	2	1.101	0.407-2.974	0.562	0%
3-year survival					
Palliative gastrectomy vs. chemotherapy only	5	0.567	0.299–1.074	0.0816	54%
Palliative gastrectomy vs. non-resectional procedures	1	-	-	-	-
Palliative gastrectomy vs. no Intervention	1	-	-	-	-

#### 2-Year Survival

Seventeen studies reported numbers surviving at 2 years following palliative surgery, non-resectional surgery, chemotherapy or no treatment. Palliative surgery was associated with an improved 2-year survival compared to non-resectional surgery (RR 0.432, 0.150–1.194; *p* = 0.44), chemotherapy (RR 0.508, 0.352–0.744; *p* = 0.04) and no treatment (RR 0.277, 0.239–0.326; *p* < 0.001) (Table 4).

#### 3-Year Survival

Eight studies reported numbers surviving at 3 years following palliative surgery, non-resectional surgery, chemotherapy or no treatment. Palliative surgery was associated with an improved 3-year survival compared to chemotherapy (RR 0.578, 0.298–1.12; *p* = 0.23) and no treatment (RR 0.225, 0.181–0.284; *p* < 0.001) (Table 4).

## Discussion

This review identifies an association between palliative gastrectomy and improved overall survival for patients with gastric cancer treated palliatively, compared to chemotherapy, non-resectional surgery and no treatment, at 1 year. After 1 year, palliative gastrectomy was not associated with a survival benefit over non-resectional surgery. Significantly, palliative gastrectomy was associated with increased morbidity compared to non-resectional surgery; however, this was not simultaneously associated with increased peri-operative mortality.

This study encompasses all relevant trials up until January 2020. Surgical techniques and oncological therapies have improved markedly during the inclusion period which extends from 1974 to 2018. Potential improvements in clinical practice may have enabled improved patient selection for gastrectomy. Improvement in surgical and oncological techniques concurrently with improved patient selection aims to optimise survival for those fit for some form of resection. Current patient selection uses criteria such as patient performance status, co-morbidity, extent of disease and importantly patient choice. The extent to which biology of the disease dictates outcome is poorly understood, however, with ongoing research into the genetics of gastric cancer [142, 143] with the potential to further refine selection in the future, further optimising outcomes [144, 145].

The study, although comprehensive, including 128 papers which included 58,675 patients did include studies from over 40 years, some of which may have limited clinical relevance; however, subgroup analyses of papers published in the last decade did not show significantly different results. The study did not incorporate outcomes for palliative gastrectomy which were combined with the outcomes of other surgical procedures such as cytoreductive surgery (CRS) which together may improve survival for those who would otherwise receive palliative oncological therapies. The precise reason for palliative surgery and the extent of disease burden was heterogeneous throughout the studies identified and the lack of current clinical guidelines or consensus on this topic makes this extremely difficult to standardise. Very few studies reported on health-related quality of life measures, following palliative gastrectomy. In an era where health research aims to re-focus on patient perceived benefits, any measured improvement in health-related quality of life could be considered more important than small improvements in quantity of life with co-morbid surgical procedures.

Challenges remain as how to determine treatment choice based on the extent of local disease and whether patients with T4b disease should receive surgery. There is significant variation in unit practice as to whether patients receive a multivisceral resection (MVR) or palliative surgery. MVR is associated with a significant morbidity and mortality in excess of the accepted risks of gastrectomy [146]. This is particularly evident when distal pancreatectomy is required to achieve an R0 resection [147]. Despite this, performing an MVR to achieve an R0 resection does provide a survival advantage and should be a potential treatment option in patients deemed sufficiently fit for surgery of this magnitude [148].

The role of surgery in metastatic gastric cancer continues to evolve as treatment options mirror treatment advances in other malignancies. Hepatectomy for colorectal liver metastasis has been show to improve survival compared to other palliative treatment options [149]. There is now evidence to demonstrate that hepatectomy for gastric cancer metastases is associated with longer median overall survival than palliative treatments for selected patients [150, 151]. Peritoneal carcinomatosis is predominantly treated with systemic chemotherapy; however, cytoreductive surgery and heated intraperitoneal chemotherapy (CRS and HIPEC) have been shown in highly selected patients to provide a survival advantage [152, 153]. Pressurised intraperitoneal aerosol chemotherapy has also been demonstrated to be safe and provides beneficial anti-tumour activity in patients with gastric cancer peritoneal carcinomatosis [154]. Although this systematic review and meta-analysis does not specifically examine the potential beneficial adjuncts to gastrectomy, it is important to identify that achieving a survival advantage with surgery may require a multi-modal approach.

It is currently not clear to what extent oncological therapies could be used in concordance with surgery and whether patients undergoing palliative resection should be offered neoadjuvant and adjuvant chemotherapy, as standard, particularly in an era where FLOT (5-fluorouracil, folinic acid, oxaliplatin, docetaxel) is becoming the gold standard of oncological treatment for patients with oesophago-gastric cancer. The REGATTA trial randomised patients to gastrectomy with D1 lymphadenectomy without any resection of metastatic lesions and adjuvant chemotherapy or chemotherapy alone and found no significant difference in overall survival [14]. Subsequently, there has been a trend away from the use of surgery in improving survival in patients who are known to have metastatic gastric cancer [155].

The AIO-FLOT 3 trial compared patients with limited metastatic disease who benefited from neoadjuvant FLOT to patients with resectable disease and to patients with extensive metastatic disease [12]. The trial identified that patients with limited metastatic disease who received neoadjuvant chemotherapy and proceeded to surgery showed a favourable survival when compared to expected survival for patients with metastatic disease. The trial did not determine the additional benefit of surgery in patients with limited metastatic disease who showed a good response to chemotherapy. Improvements in chemotherapy in conjunction with improving surgical techniques inclusive of a D2 gastrectomy and metastatectomy may provide improved survival for patients who previously may have been palliated.

Oncological therapies continue to develop and immunotherapy is increasingly playing a role in gastric cancer as is evident with HER2 positive tumours and the use of trastuzumab [156]. Further studies continue into the importance of HER-2 blockade in the form of trastuzamab and pertuzamab in conjunction with FLOT in the Petrarca Trial which is yet to report [157]. Increasingly immunotherapy trials continue to examine the benefits of PD1/PD-L1 and CTLA4 blockade and will likely be incorporated into the treatment pathways of advanced gastric cancer [158–160].

## Conclusions

Palliative gastrectomy is associated with significant morbidity over and above non-resectional palliative surgery and gastrectomy for curative intent. Palliative gastrectomy may offer an early survival advantage compared to oncological therapies given in isolation; however, this does not extend beyond a couple of years and may well result from patient selection biases. Further research into the biology of gastric cancer and improved techniques for patient selection are required to improve overall survival for patients with palliative gastric cancer.

## Electronic supplementary material

ESM 1 (DOCX 1622 kb).
