# Supplementary material for: Peri-operative Outcomes and Survival Following Palliative Gastrectomy for Gastric Cancer: a Systematic Review and Meta-analysis
Source: J Gastrointest Cancer. 2020 Sep 22;52(1):41–56. doi: 10.1007/s12029-020-00519-4 (PMC7900337; doi:10.1007/s12029-020-00519-4)

**Supplementary table 1 – Literature search terms**

| 1 | palliative gastrectomy.ti,ab. | 312 |
| --- | --- | --- |
| 2 | palliative total gastrectomy.ti,ab. | 48 |
| 3 | palliative subtotal gastrectomy.ti,ab. | 21 |
| 4 | palliative resection.ti,ab. | 2234 |
| 5 | 1 or 2 or 3 or 4 | 2581 |
| 6 | exp Stomach Neoplasms/ | 221931 |
| 7 | gastric cancer.ti,ab. | 138103 |
| 8 | gastric adenocarcinoma.ti,ab. | 16016 |
| 9 | stomach cancer.ti,ab. | 13760 |
| 10 | 6 or 7 or 8 or 9 | 261158 |
| 11 | exp INTRAOPERATIVE COMPLICATIONS/ or exp POSTOPERATIVE COMPLICATIONS/ | 1156960 |
| 12 | complications.ti,ab. | 1785921 |
| 13 | Post-operative complications.ti,ab. | 17738 |
| 14 | exp HOSPITAL MORTALITY/ or exp MORTALITY/ | 1378262 |
| 15 | mortality.ti,ab. | 1939489 |
| 16 | exp DISEASE-FREE SURVIVAL/ or exp SURVIVAL ANALYSIS/ or exp SURVIVAL/ or exp SURVIVAL RATE/ | 1398135 |
| 17 | overall survival.ti,ab. | 634483 |
| 18 | disease-free survival.ti,ab. | 100973 |
| 19 | exp Patient Reported Outcome Measures/ | 13943 |
| 20 | exp "Quality of Life"/ | 647781 |
| 21 | quality of life.ti,ab. | 758027 |
| 22 | 11 or 12 or 13 or 14 or 15 or 16 or 17 or 18 or 19 or 20 or 21 | 6677652 |
| 23 | 5 and 10 and 22 | 579 |
| 24 | palliation.ti,ab. | 36141 |
| 25 | palliative.ti,ab. | 173076 |
| 26 | non-curative gastrectomy.ti,ab. | 42 |
| 27 | palliative surgery.ti,ab. | 4814 |
| 28 | palliative treatment.ti,ab. | 16146 |
| 29 | adenocarcinoma of gastric antrum.ti,ab. | 10 |
| 30 | adenocarcinoma of the gastric antrum.ti,ab. | 39 |
| 31 | noncurative.ti,ab. | 1550 |
| 32 | gastric carcinoma.ti,ab. | 28755 |
| 33 | 5 or 24 or 25 or 26 or 27 or 28 or 31 | 201744 |
| 34 | 10 or 29 or 30 or 32 | 265350 |
| 35 | 22 and 33 and 34 | 4202 |

**Supplementary table 1 – Newcastle Ottawa Scores for studies**

| **Study Names** | **S** | **C** | **O** | **T** | **Study Names** | **S** | **C** | **O** | **T** |
| --- | --- | --- | --- | --- | --- | --- | --- | --- | --- |
| Lulu 1974 | 4 | 0 | 3 | 7 | Lello 2007 | 4 | 2 | 3 | 9 |
| Zacho 1974 | 3 | 0 | 3 | 6 | Mizutani 2007 | 4 | 2 | 2 | 8 |
| Zwaveling 1976 | 4 | 0 | 2 | 6 | Nazli 2007 | 4 | 2 | 2 | 8 |
| Nelson 1982 | 4 | 0 | 2 | 6 | Kim 2008 | 4 | 2 | 3 | 9 |
| Yap 1982 | 4 | 0 | 3 | 7 | Pacelli 2008 | 4 | 0 | 3 | 7 |
| Choi 1982 | 3 | 0 | 3 | 6 | Du 2008 | NC |  |  |  |
| Meijer 1983 | 4 | 0 | 2 | 6 | Lin 2008 | 4 | 1 | 3 | 8 |
| Yan 1985 | 4 | 0 | 3 | 7 | Park 2009 | 4 | 2 | 3 | 9 |
| Cunningham 1987 | 4 | 1 | 3 | 8 | Lupascu 2010 | 4 | 0 | 2 | 6 |
| Bozzetti 1987 | 4 | 0 | 2 | 6 | Huang 2010 | 4 | 2 | 2 | 8 |
| de 1988 | 4 | 0 | 2 | 6 | Hioki 2010 | 4 | 2 | 3 | 9 |
| Butler 1989 | 3 | 1 | 3 | 7 | Sah 2010 | 4 | 2 | 3 | 9 |
| Haugstvedt 1989 | 4 | 2 | 3 | 9 | Ozer 2010 | 4 | 1 | 2 | 7 |
| Carmalt 1990 | 4 | 0 | 3 | 7 | Li 2010 | 4 | 1 | 2 | 7 |
| Habu 1990 | 4 | 1 | 3 | 8 | Xue 2010 | 4 | 2 | 3 | 9 |
| Nakajima 1991 | 4 | 0 | 1 | 5 | Turanli 2010 | 4 | 2 | 2 | 8 |
| Yonemura 1991 | 3 | 0 | 2 | 5 | Schauer 2011 | 4 | 2 | 3 | 9 |
| Monson 1991 | NC |  |  |  | Al-Amawi 2011 | 4 | 0 | 2 | 6 |
| Maehara 1992 (1) | 4 | 2 | 3 | 9 | Tanizawa 2011 | 4 | 0 | 3 | 7 |
| Maehara 1992 | 4 | 0 | 2 | 6 | Zhang 2011 | 4 | 0 | 3 | 7 |
| Huguier 1992 | 4 | 0 | 2 | 6 | Izuishi 2011 | 4 | 2 | 3 | 9 |
| Maehara 1992 | 4 | 0 | 2 | 6 | Lai 2011 | 4 | 2 | 3 | 9 |
| Baba 1992 | 4 | 2 | 3 | 9 | Miki 2012 | 4 | 2 | 2 | 8 |
| Geoghegan 1993 | 4 | 0 | 2 | 6 | Kokkola 2012 | 4 | 2 | 3 | 9 |
| Ti 1993 | NC |  |  |  | Shim 2012 | 2 | 1 | 2 | 5 |
| Crookes 1995 | 4 | 0 | 2 | 6 | Alonso-Larraga 2012 | 4 | 0 | 3 | 7 |
| Chow 1995 | 4 | 2 | 3 | 9 | Tokunaga 2012 | 4 | 1 | 3 | 8 |
| Arak 1996 | 4 | 2 | 2 | 8 | Amaral 2012 | 3 | 0 | 3 | 6 |
| Saito 1996 |  |  |  |  | Naka 2012 | 4 | 1 | 3 | 8 |
| Cenitagoya 1998 | 4 | 1 | 3 | 8 | Chang 2012 | 4 | 2 | 2 | 8 |
| Kikuchi 1998 | 4 | 2 | 3 | 9 | Kang 2013 | 4 | 2 | 2 | 8 |
| Sanchez-Bueno 1998 | 4 | 2 | 3 | 8 | Keranen 2013 | 4 | 2 | 3 | 9 |
| Piso 1998 | NC |  |  |  | He 2013 | 4 | 2 | 3 | 9 |
| Ouchi 1998 | 4 | 2 | 2 | 8 | Ikeguchi 2013 | 4 | 2 | 3 | 9 |
| Piso, | NC |  |  |  | Xia 2014 | 3 | 2 | 3 | 8 |
| Lo 1999 | 4 | 2 | 2 | 8 | Kwon 2014 | 4 | 2 | 2 | 8 |
| Doglietto 1999 | 4 | 2 | 2 | 8 | Zeeneldin 2014 | 4 | 2 | 3 | 9 |
| Llanos 1999 | 4 | 0 | 2 | 6 | Zeng 2014 | 4 | 2 | 3 | 9 |
| Saidi 1999 | 4 | 0 | 3 | 7 | Jeong 2014 | 4 | 2 | 3 | 9 |
| Doglietto 2000 | 4 | 2 | 2 | 8 | Kim 2014 | 4 | 1 | 3 | 8 |
| Ikeguchi 2001 | 4 | 2 | 2 | 8 | da 2015 | 4 | 2 | 3 | 9 |
| Hanazaki 2001 | 4 | 0 | 2 | 6 | Matsumoto 2015 | 4 | 2 | 3 | 9 |
| Dhar 2001 | 4 | 2 | 3 | 9 | Yao 2015 | 4 | 0 | 3 | 7 |
| Fujisaki 2001 | 4 | 2 | 2 | 8 | Yang 2015 | 4 | 2 | 2 | 8 |
| Bonenkamp 2001 | 4 | 1 | 3 | 8 | Ebinger 2016 | 4 | 2 | 2 | 8 |
| Liu 2002 | 3 | 1 | 3 | 7 | Dong 2016 | 4 | 0 | 2 | 6 |
| Wang 2002 | 4 | 2 | 3 | 9 | Coimbra 2016 | 4 | 2 | 2 | 8 |
| Collard 2003 | 3 | 2 | 3 | 8 | Chiu 2016 | 4 | 2 | 2 | 8 |
| Yoshikawa 2003 | 4 | 2 | 2 | 8 | Musri 2016 | 4 | 2 | 3 | 9 |
| Gill, | 3 | 2 | 2 | 7 | Ikeguchi 2016 | 4 | 0 | 3 | 7 |
| Kobayashi 2004 | 4 | 2 | 2 | 8 | Nie 2016 | 4 | 2 | 3 | 9 |
| Moriwaki 2004 | 4 | 0 | 3 | 7 | Tokunaga 2016 | 4 | 2 | 3 | 9 |
| Kahlke 2004 | 4 | 0 | 2 | 6 | Al-Batran 2017 | RCT |  |  |  |
| Medina-Franco | 4 | 0 | 3 | 7 | Fujitani 2017 | 4 | 0 | 3 | 7 |
| Zhang 2004 | 4 | 2 | 3 | 9 | Hsu 2017 | 4 | 2 | 1 | 7 |
| Gorbunov 2005 | 4 | 1 | 2 | 7 | Fornaro 2017 | 4 | 2 | 2 | 8 |
| Kunisaki 2005 | 3 | 1 | 3 | 7 | Yuan 2017 | 4 | 2 | 3 | 9 |
| Alici 2006 | 4 | 2 | 3 | 9 | Fukuchi 2018 | 4 | 2 | 3 | 9 |
| Saidi 2006 | 4 | 0 | 2 | 6 | Warschkow 2018 | 4 | 2 | 2 | 8 |
| Samarasam 2006 | 4 | 0 | 3 | 7 | Picado 2018 | 4 | 2 | 2 | 8 |
| Onate-Ocana 2007 | 4 | 0 | 2 | 6 | Yuan 2018 | 4 | 2 | 2 | 8 |
| Nazli 2007 | 4 | 0 | 2 | 6 | Yang 2019 | 3 | 2 | 3 | 8 |
| Lim 2007 | 4 | 2 | 3 | 9 | Omori 2019 | 3 | 2 | 2 | 7 |
|  |  |  |  |  | Matsubara 2019 | 2 | 2 | 2 | 6 |

**Abbreviations: S: Selection, C: Comparability, O: Outcome, T:Total out of 9, NC: Non-comparative cohort**

**Supplementary Figure 1 – Forest plots showing odds ratios and 95% confidence intervals for palliative gastrectomy compared to curative surgery for A) overall complications B) major complications C) anastomotic leak D) mortality**


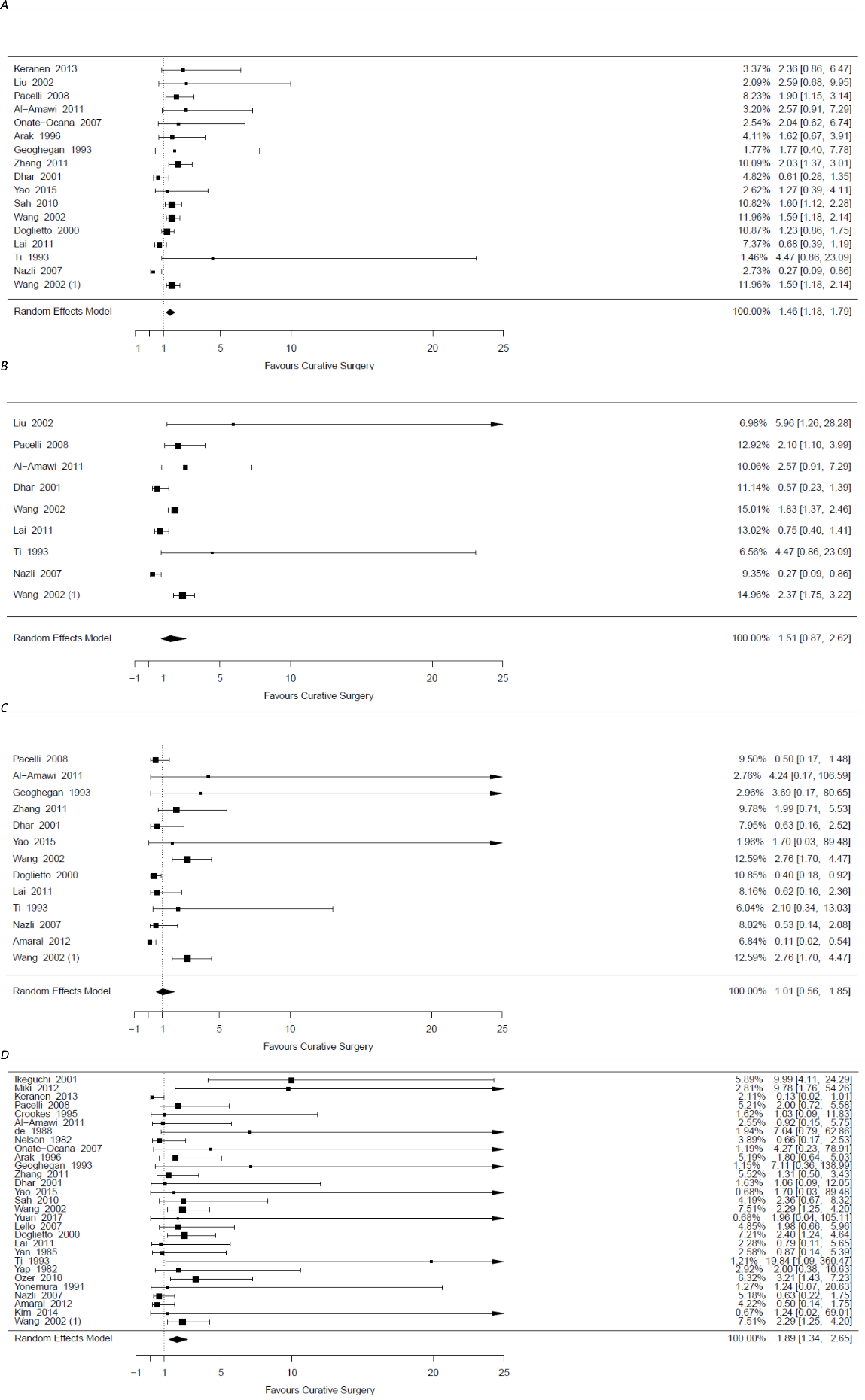


**Supplementary Figure 2 – Forest plots showing odds ratios and 95% confidence intervals for palliative gastrectomy compared to non tumour resective surgery for A) overall complications B) major complications C) anastomotic leak D) mortality**


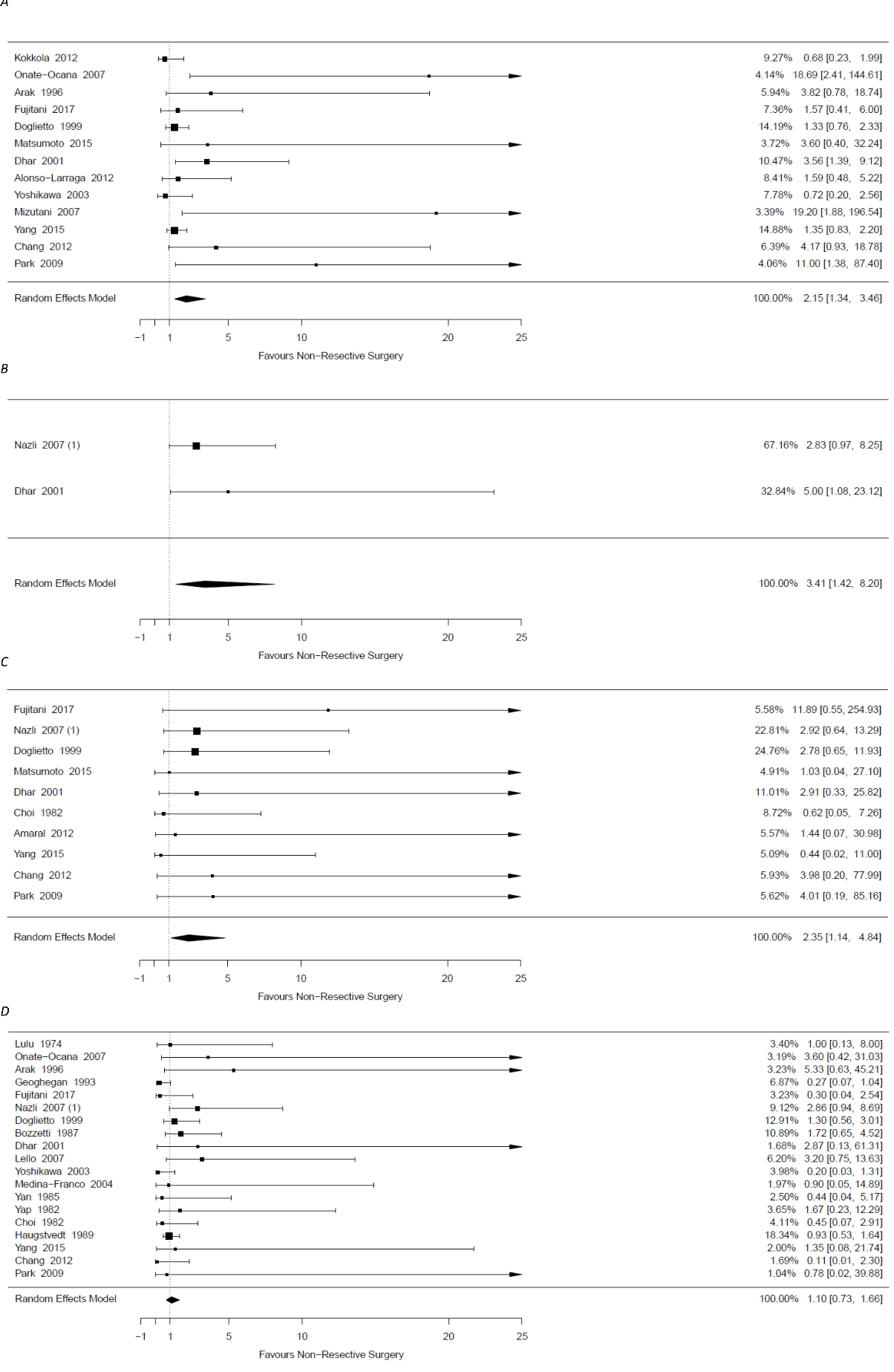


**Supplementary Figure 3 – Forest plots showing relative risk of survival at 1 year for palliative gastrectomy compared to A) Chemotherapy only B) Non tumour resectional surgery C) No intervention**


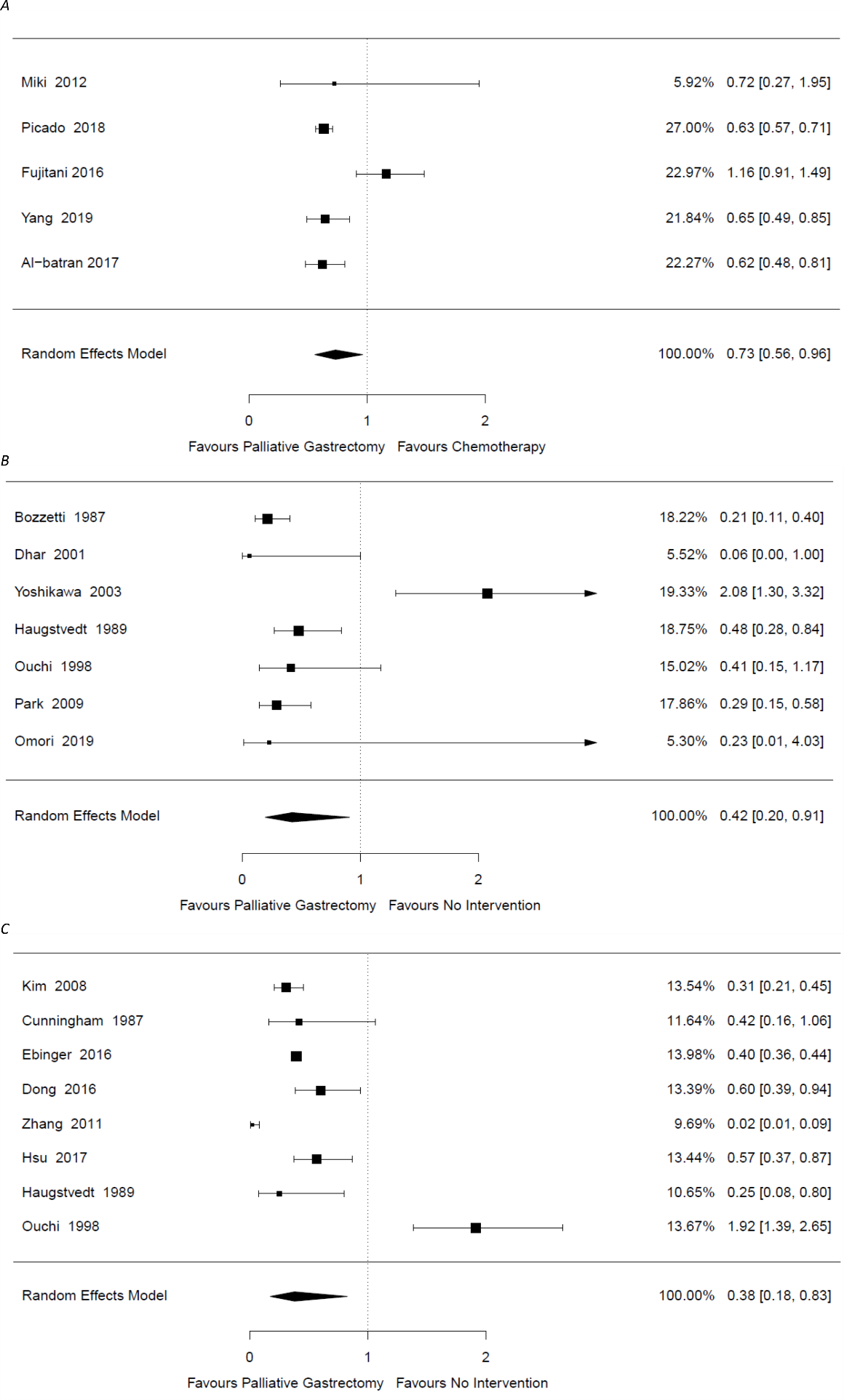


**Supplementary Figure 4 – Forest plots showing relative risk of survival at 2 years for palliative gastrectomy compared to A) Chemotherapy only B) Non tumour resectional surgery C) No intervention**


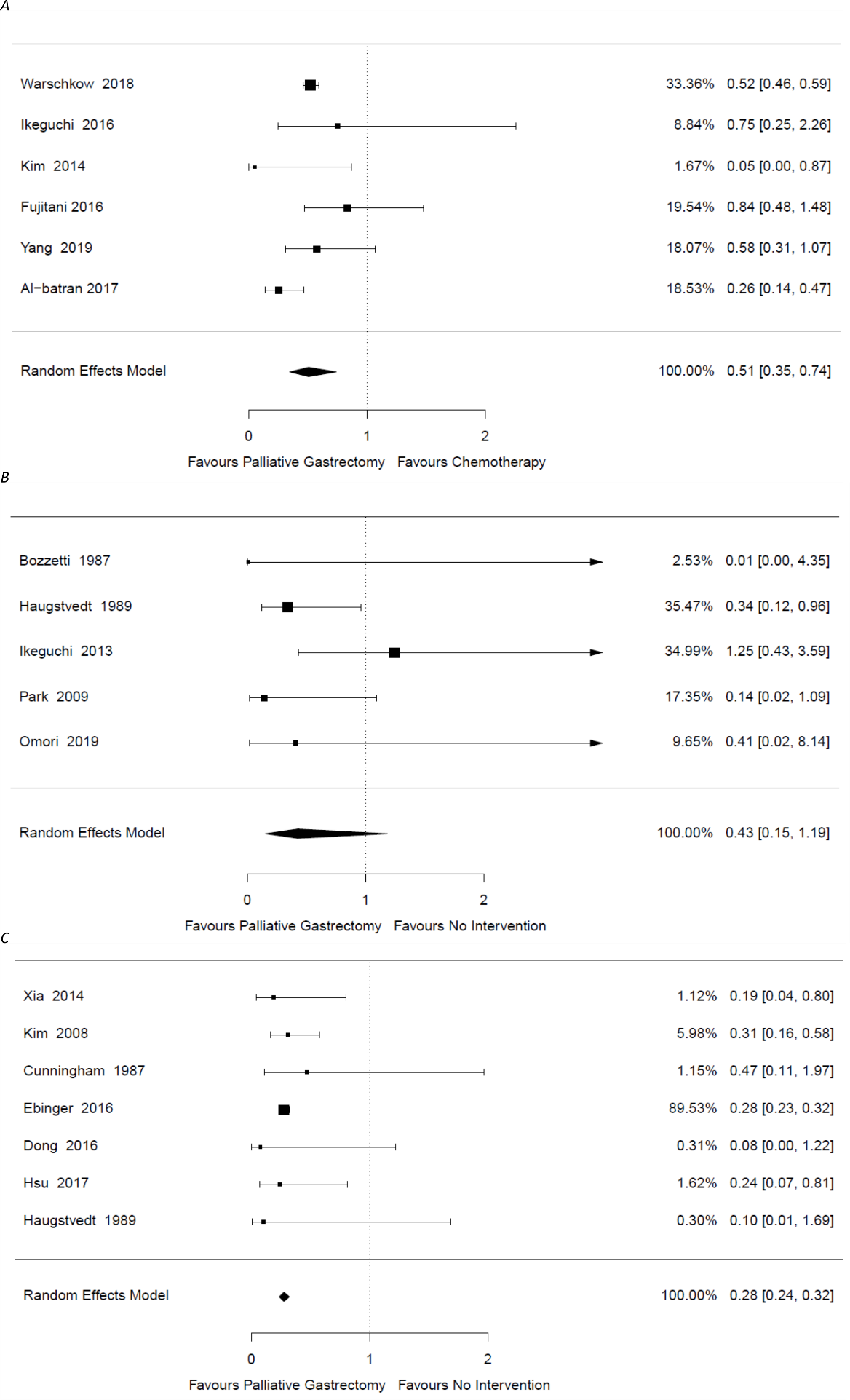


**Supplementary Figure 5 – Forest plots showing relative risk of survival at 2 years for palliative gastrectomy compared to A) Chemotherapy only B) No intervention**


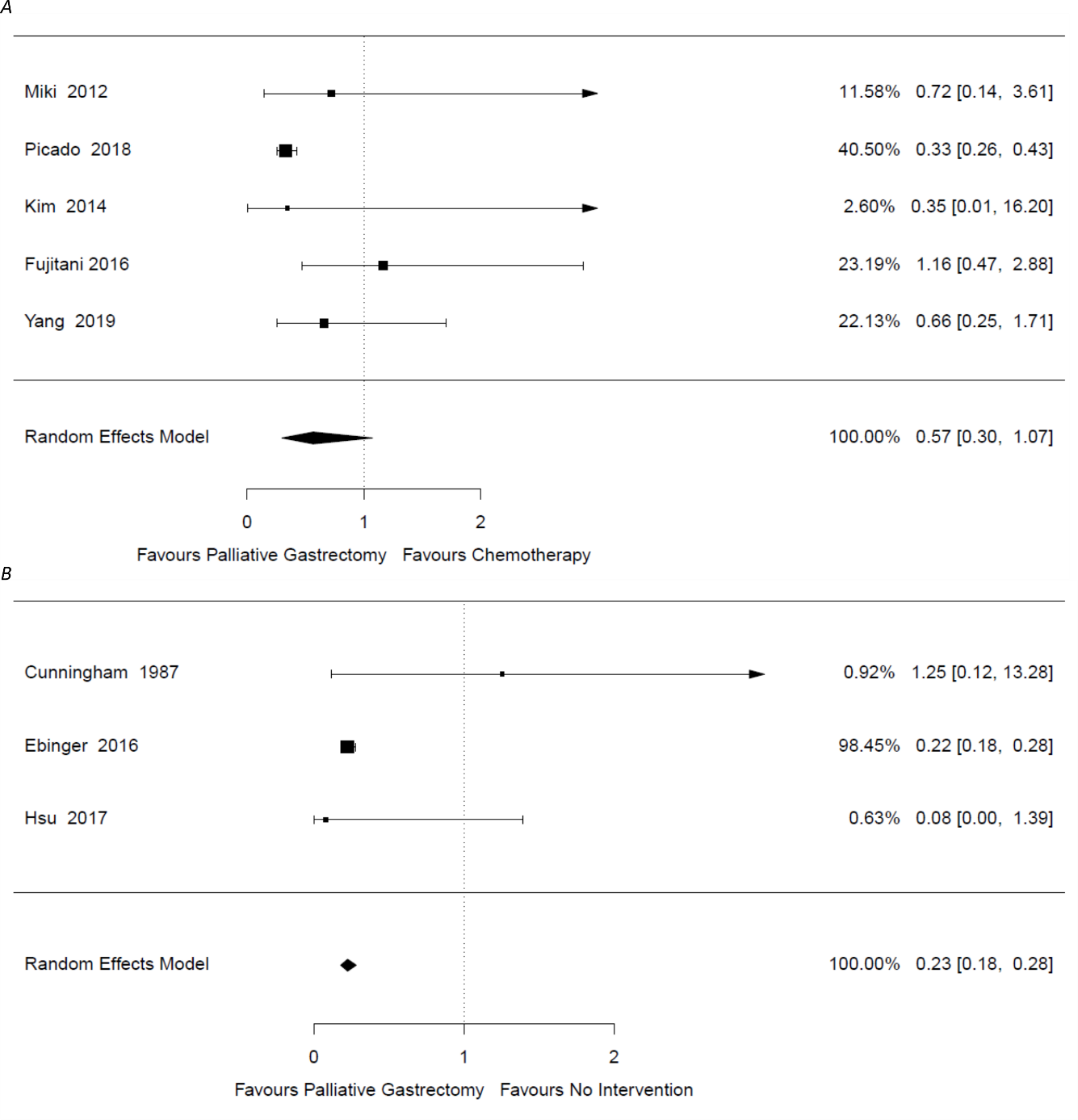

Supplement: Supplementary file 1 — (DOCX 1622 kb). [file 12029_2020_519_MOESM1_ESM.docx]
